# Effective implicit finite‐difference method for sensitivity analysis of stiff stochastic discrete biochemical systems

**DOI:** 10.1049/iet-syb.2017.0048

**Published:** 2018-08-01

**Authors:** Monjur Morshed, Brian Ingalls, Silvana Ilie

**Affiliations:** ^1^ Department of Applied Mathematics University of Waterloo Waterloo Ontario N2L 3G1 Canada; ^2^ Department of Mathematics Ryerson University Toronto Ontario M5B 2K3 Canada

**Keywords:** biochemistry, sensitivity analysis, stochastic processes, cellular biophysics, probability, fluctuations, master equation, reaction kinetics, finite difference methods, effective implicit finite‐difference method, sensitivity analysis, stiff stochastic discrete biochemical systems, cellular processes, mathematical modelling, deterministic differential equation models, inherently probabilistic‐stochastic models, random fluctuations, Chemical Master Equation, biochemical kinetics, kinetic parameter estimation, systems dynamics, CME models, biochemical reaction systems, finite‐difference approximations, adaptive implicit tau‐leaping strategies, computational efficiencies

## Abstract

Simulation of cellular processes is achieved through a range of mathematical modelling approaches. Deterministic differential equation models are a commonly used first strategy. However, because many biochemical processes are inherently probabilistic, stochastic models are often called for to capture the random fluctuations observed in these systems. In that context, the Chemical Master Equation (CME) is a widely used stochastic model of biochemical kinetics. Use of these models relies on estimates of kinetic parameters, which are often poorly constrained by experimental observations. Consequently, sensitivity analysis, which quantifies the dependence of systems dynamics on model parameters, is a valuable tool for model analysis and assessment. A number of approaches to sensitivity analysis of biochemical models have been developed. In this study, the authors present a novel method for estimation of sensitivity coefficients for CME models of biochemical reaction systems that span a wide range of time‐scales. They make use of finite‐difference approximations and adaptive implicit tau‐leaping strategies to estimate sensitivities for these stiff models, resulting in significant computational efficiencies in comparison with previously published approaches of similar accuracy, as evidenced by illustrative applications.

## 1 Introduction

The dynamics of reaction networks in living organisms have been extensively studied in Systems Biology [1, 2]. Dynamics in such complex networks is driven by random thermal agitation. Consequently, stochastic modelling is a preferred approach for studying the behaviour of these biochemical reaction networks [3–7].

The most widely used stochastic model for describing spatially homogeneous biochemical reaction dynamics is the Chemical Master Equation (CME). State trajectories for the CME can be simulated via Gillespie's [8, 9] stochastic simulation algorithm (SSA), a Monte Carlo method. When describing systems with large molecular populations or widely varying time‐scales, Gillespie's algorithm is computationally expensive. To reduce computational costs, Gillespie [10] introduced a variant of his algorithm method called tau‐leaping, in which time steps are selected dynamically to avoid exhaustive calculations, with tolerable loss of accuracy.

The time steps for the explicit tau‐leaping strategy are limited to the fastest mode. Consequently, this strategy is not suitable for stiff biochemical systems. Specifically, the explicit tau‐leaping technique generalises the explicit Euler method for ordinary differential equations to discrete stochastic systems. Similar to Euler's method, when taking large time steps, the explicit tau‐leaping scheme shows instability for very stiff biochemical systems. Rathinam *et al.* [11] proposed the implicit tau‐leaping technique to overcome the stability issues of the explicit strategy, thus allowing larger time steps. The implicit tau‐leaping scheme employs larger stepsizes than the explicit tau‐leaping strategy for stiff discrete stochastic systems while producing a solution of similar accuracy as the explicit tau‐leaping scheme for the slow manifold and for the mean of the fast variables on the slow manifold [11]. The implicit tau‐leaping may damp the noise for some systems, as illustrated in [11] for systems reaching steady state.

Sensitivity analysis, which describes how model parameters related to system dynamics, is a key tool for model development and analysis [12]. Sensitivity analyses can be characterised as global (sampling over a broad region of the parameter space) or local (describing behaviour in the neighbourhood of a nominal operating point). Global approaches are computationally demanding because they require many samples from a (typically high‐dimensional) parameter space [13]. In contrast, local sensitivity analysis involves only small perturbations about a nominal parameterisation. Sensitivity analysis is a powerful tool for investigating the dynamic properties of a biochemical system. A small sensitivity reflects robustness, while large sensitivities can indicate parameters that have significant effects on outputs of interest. For biochemical reaction systems, the parameters of interest include the initial conditions, kinetic rate constants of the reactions, and environmental parameters (such as temperature). For deterministic models, local sensitivity analysis is rarely computationally demanding [14]. However, the corresponding analysis of stochastic models requires large ensembles of simulated sample paths.

Local sensitivity coefficients are usually determined as finite differences. The sensitivity of the expected value of an output *f* of a stochastic system to a parameter *c* can be described as $[E(f(X^{c+h}(t)))-E(f(X^{c}(t)))]/h$, where $X^{c}(t)$ represents the state of the system at time *t* and parameter *c*. The perturbation *h* is small compared to the nominal value of parameter *c*. To determine the sensitivity coefficient, two ensembles of sample paths are generated, corresponding to the parameter values *c* and *c* + *h*. A number of approaches to the calculation of such finite‐difference estimators have appeared recently in the literature, including the common random number (CRN) and common reaction path (CRP) approaches [15], the coupled finite‐difference (CFD) scheme [16], and the coupled tau‐leaping (CTL) algorithm [17].

For each of these sensitivity methods, sample paths of the nominal ($X^{c}$) and perturbed $\left(X^{c+h}\right)$ systems are simulated, using a common random seed to reduce variance. Anderson's CFD method, in which trajectories of the two systems are strongly coupled, was found to produce the smallest estimator variance of these approaches [18] and can be used effectively for non‐stiff models.

For stiff models, exact stochastic simulation is computationally expensive. The CTL [17] scheme was introduced to address this issue by applying an explicit tau‐leaping in the context of trajectory coupling. Because it uses the explicit tau‐leaping strategy, this method works well for moderately stiff problems.

In this paper, we propose a novel finite‐difference method for estimating sensitivities of stiff stochastic models of biochemical reaction networks. This approach, described below as the Coupled implicit‐Tau (CIT) algorithm, makes use of an implicit‐tau leaping scheme to efficiently generate sample trajectories of systems in which some reactions are in effective equilibrium. This adaptive time leaping bears similarities to an algorithm proposed by Cao *et al.* [19]. Similar to the CFD method mentioned earlier, an important property of the τ‐leaping scheme for perturbation analysis is that the nominal and perturbed systems are strongly coupled. A similar method was developed by Anderson and Higham [20] for continuous time Markov Chains. This coupling reduces the variance in the finite‐difference estimator, allowing for a more precise measure of sensitivity.

The remaining of this paper is organised as follows. Section 2 gives an introduction to stochastic modelling and simulation of well‐stirred biochemical systems. In Section 3, we discuss the finite‐difference approximation of sensitivity for stochastic models of biochemical networks. The new CIT algorithm is presented in Section 4. Numerical tests comparing the CIT and the CFD methods are given in Section 5. Finally, we summarise our conclusions in Section 6.

## 2 Stochastic biochemical kinetics

We consider a well‐stirred system of biochemical reactions kept in a constant volume, at a constant temperature. The chemical species $S_{1},\ldots ,S_{N}$ are involved in the biochemical reactions $R_{1},\ldots ,R_{M}$. The state vector $X(t)=(X_{1}(t),\ldots ,X_{N}(t)){\;}^{T}$ indicates the species abundance at each time *t*.

Each reaction $R_{j}$ is characterised by a propensity function $a_{j}(\cdot )$ and a state change (stoichiometry) vector $\nu_{j}$. The propensity $a_{j}(x)$ is defined as follows: $a_{j}(x)dt$ is the probability that a single reaction $R_{j}$ fire in the interval [t,t+dt), provided that X(t)=x. The stoichiometry vector $\nu_{j}$ describes the change in molecule abundance as a consequence of reaction $R_{j}$ occurring: if the system is in the state x, and then a reaction $R_{j}$ occurs, the system state becomes $x+\nu_{j}$. The matrix $v=(\nu_{j}){\;}_{j=1,\ldots ,M}$ is the stoichiometry matrix.

The conditional probability of the system being in state x at time *t*, X(t)=x, provided that $X(t_{0})=x_{0}$ is denoted by $P(x,t|x_{0},t_{0})$. This probability obeys:

(1) $$\begin{array}{rl} \frac{dP(x,t|x_{0},t_{0})}{dt} & =\sum_{j=1}^{M}[a_{j}(x-\nu_{j})P(x-\nu_{j},t|x_{0},t_{0})\\ & \;-a_{j}(x)P(x,t|x_{0},t_{0})] \end{array}$$

which is known as the Chemical Master Equation.

### 2.1 Stochastic simulation algorithm

Gillespie proposed a Monte Carlo method, the SSA [9], for simulating sample paths with a probability in exact agreement with the solution of the CME. This algorithm is summarised below:

Initialise the system state $X(t_{0})=x_{0}$ at $t_{0}=0$.

while *t* < *T*

1. Calculate the propensities $\{a_{k}(X(t))\}{\;}_{k=1}^{M}$ and set $a_{0}(X(t)):=\sum_{k=1}^{M}a_{k}(X(t))$.
2. Sample $\xi_{1}$ and $\xi_{2}$ from a uniform distribution on [0, 1], denoted by *U*(0, 1).
3. Set *j*, the smallest integer satisfying $\sum_{k=1}^{j}a_{k}(X(t))>\xi_{1}a_{0}(X(t))$, be the index of the next reaction.
4. Set $\tau =\ln(1/\xi_{2})/a_{0}(X(t))$ as the time for the next reaction.
5. Update $X(t+\tau )=X(t)+\nu_{j}$ and t=t+τ.
  end while

### 2.2 Tau‐leaping methods

Exact Monte Carlo simulation algorithms [8, 9, 21] for the CMEs are often computationally expensive for problems of practical interest. An approximate strategy that reduces the computational cost of solving the CME is the tau‐leaping method, proposed by Gillespie [10]. In the tau‐leaping scheme, the system is advanced over many reactions with a preassigned stepsize τ. The step τ must satisfy the leap condition, which states that the propensities $a_{j}(X(t))$ remain approximately constant over [t,t+τ]. When the leap condition is met, the number of reactions $R_{j}$ occurring between [t,t+τ] can be approximated by a Poisson random variable, $P_{j}(a_{j}(x),\tau )$, with mean and variance a(x)τ, when X(t)=x. Then the system state in may be calculated as

(2) $$X(t+\tau )=x+\sum_{j=1}^{M}\nu_{j}P_{j}(a_{j}(x),\tau ),$$

given that X(t)=x. Here $P_{j}$, with 1≤j≤M, are independent Poisson random variables. Formula (2) is called the (explicit) *tau‐leaping method* and was introduced by Gillespie [10].

Many biochemical systems arising in applications are stiff, displaying both slow and fast dynamics, with the fast modes being stable. The explicit tau‐leaping strategy is impractical for stiff systems, as its time step is limited to the fastest mode. To deal with this challenge, Rathinam *et al.* [11] proposed the *implicit tau‐leaping method*. The implicit tau‐leaping technique overcomes the stability issue of the explicit strategy, allowing larger steps in time. Consequently, for stiff stochastic biochemical systems, it is more efficient than the explicit method while maintaining a similar accuracy. In fact, the scheme is semi‐implicit, being implicit only in the mean part of each term $P_{j}(a_{j},\tau )$, i.e. $a_{j}\tau$. If X(t)=x, the implicit tau‐leaping method updates the system state as

(3) $$\begin{array}{rl} X(t+\tau ) & =x+\sum_{j=1}^{M}\nu_{j}a_{j}(X(t+\tau ))\tau \\ & \;+\sum_{j=1}^{M}\nu_{j}\left[P_{j}(a_{j}(x),\tau )-a_{j}(x)\tau \right]. \end{array}$$

### 2.3 Stepsize selection for implicit tau‐leaping

A reversible reaction can come to equilibrium between reactants and products. When this occurs for some reversible reactions while the rest of the system is still undergoing significant variation, the system is said to be in *partial equilibrium*. Partial equilibrium occurs when the forward and backward propensities of a reversible reaction are approximately equal, i.e. their difference is much smaller than the propensities themselves. More precisely, if the propensities of the reversible reactions are denoted by $a_{+}(x)$ and $a_{-}(x)$, the partial equilibrium condition is

(4) $$|a_{+}(x)-a_{-}(x)|\leq \delta \min\{a_{+}(x),a_{-}(x)\},$$

for some small quantity δ>0. (In the implementations below we used δ=0.05.)

We make use of the stepsize selection strategy introduced by Cao *et al.* [19]. For those reactions that are not in partial equilibrium, we demand that the mean and variance of each reactant population $X_{i}$ should satisfy

(5) $$\left|X_{i}(t+\tau )-x_{i}|\leq \max\{\varepsilon x_{i}/g_{i},1\right\}$$

where ε is the given tolerance. The scalar $g_{i}$ represents the highest order in which species $S_{i}$ reacts (see [22] for further details)

(A) If $\psi_{i}=1$, take $g_{i}=1$
(B) If $\psi_{i}=2$, take $g_{i}=2$, unless the left hand side of the reaction is $S_{i}+S_{i}$, in which case take $g_{i}=\left(2+(1/(x_{1}-1))\right).$
(C) If $\psi_{i}=3$, take $g_{i}=3$, unless the left hand side of the reaction is $S_{i}+S_{i}+S_{j}$, in which case take $g_{i}=(3/2)\left(2+(1/(x_{i}-1))\right),$ or the reaction is $S_{i}+S_{i}+S_{i}$, in which case take $g_{i}=\left(3+(1/(x_{i}-1))+(2/(x_{i}-2))\right).$

Following [22], we arrive at an efficient implementation of this leap condition by classifying all reactions that are not in partial equilibrium as critical or non‐critical, as follows. We begin by specifying the value of a control parameter, $n_{c}$. Typically $n_{c}\in [2,20]$, see [22]. If some molecular amounts approach zero during the integration, then there is a trade‐off between maintaining these population numbers positive and the efficiency of the algorithm. A larger $n_{c}$ decreases the chance of negative population numbers while reducing the efficiency of the method. If a reactant is within $n_{c}$ firings of producing a zero population, it is called a *critical reaction*. Let us denote by $J_{\text{cr}}$, $J_{\text{ncr}}$, and $J_{\text{ne}}$ the set of indices of critical, non‐critical and not in partial equilibrium reactions, respectively. Let $J_{\text{necr}}=J_{\text{ncr}}⋂J_{\text{ne}}$ be the index set of the reaction channels that are non‐critical and not in partial equilibrium. Also, $I_{\text{ncr}}$ denotes the set of indices of species that are the reactants of non‐critical reactions.

*Non‐critical, not in partial equilibrium reactions:* The implicit tau‐leaping method is applied to the leap condition (5) implemented using the time‐step τ:

(6) $$\tau_{1}=\underset{i}{\min}\left\{\frac{\max\{\varepsilon x_{i}/g_{i},1\}}{\left|{\hat{\mu }}_{i}(x)\right|},\frac{\max{\left\{\varepsilon x_{i}/g_{i},1\right\}}^{2}}{{\hat{\delta }}_{i}^{2}(x)}\right\},$$

with the auxiliary quantities

(7) $${\hat{\mu }}_{i}(x)=\sum_{j\in J_{\text{necr}}}\nu_{ij}a_{j}(x),$$

(8) $${\hat{\delta }}_{i}^{2}(x)=\sum_{j\in J_{\text{necr}}}\nu_{ij}^{2}a_{j}(x).$$

*Critical reactions:* These reactions are implemented on the nominal or perturbed trajectory using the SSA, one critical reaction at a time. For advancing a critical reaction on the nominal trajectory two samples of the uniform random variable on the unit interval, *U*(0,1) are computed, $\xi_{1}^{\left(c\right)}$ and $\xi_{2}$, along with the sum of all propensities of the critical reactions $a_{0}^{\text{cr},(c)}(X^{c}))$. Then, the index of the next critical reaction of the nominal trajectory is the smallest integer $j_{\text{cr}}$ satisfying

(9) $$\sum_{\ell \leq j_{\text{cr}},\ell \in J_{\text{cr}}}a_{\ell }^{c}(X^{\left(c\right)})>\xi_{2}a_{0}^{\text{cr},(c)},$$

and the time to the next reaction is

(10) $$\tau_{2}^{\left(c\right)}=(1/a_{0}^{\text{cr},(c)}(X^{c}))\ln(1/\xi_{1}^{\left(c\right)}).$$

Similarly, for finding the next critical reaction on the perturbed trajectory, two samples of the uniform random variable on the unit interval are computed, $\xi_{1}^{\left(c+h\right)}$ and $\xi_{2}$, and the sum of all propensities of the critical reactions $a_{0}^{\text{cr},(c+h)}(X^{c+h}))$. The next reaction on the perturbed trajectory has index $j_{\text{cr}}$ given by the smallest integer obeying

(11) $$\sum_{\ell \leq j_{\text{cr}},\ell \in J_{\text{cr}}}a_{\ell }^{c+h}(X^{\left(c+h\right)})>\xi_{2}a_{0}^{\text{cr},(c+h)}$$

and occurs after a time step

(12) $$\tau_{2}^{\left(c+h\right)}=(1/a_{0}^{\text{cr},(c+h)}(X^{c+h}))\ln(1/\xi_{1}^{\left(c+h\right)}).$$

### 2.4 Finite‐difference methods for sensitivity analysis of stochastic biochemical systems

Each of the techniques described above for approximating parametric sensitivities for stochastic discrete models of biochemical kinetics involves the forward finite‐difference estimator $[E(f(X^{c+h}(t)))-E(f(X^{c}(t)))]/h$, where *h* represents a perturbation, *c* is the parameter of interest, *X* is the state of the chemical reaction system, and *f* is the output of interest. This finite‐difference estimator approximates the local sensitivity of the expected value of the quantity $f(X^{c}(t))$ with respect to a parameter *c*, given a polynomial function *f*. (Note that higher‐order moments can be determined by appropriate combinations of expected sensitivities.)

Among the existing finite‐difference strategies [15, 16] for stochastic discrete models of biochemical kinetics, the CFD method due to Anderson in [16] was shown to produce the smallest estimator variance. It simulates the coupled trajectories with the next reaction method. This sensitivity estimator is based on the following tight coupling between the nominal process, $X^{c}(t)$, and the perturbed process, $X^{c+h}(t)$,

(13) $$\begin{array}{rl} X^{c}(t) & =X^{c}(0)+\sum_{j=1}^{M}\nu_{j}Y_{j,1}\left(\int_{0}^{t}m_{j,c,h}(s)\;ds\right)\\ & \;+\sum_{j=1}^{M}\nu_{j}Y_{j,2}\left(\int_{0}^{t}a_{j}^{c}(X^{c}(s))-m_{j,c,h}(s)\;ds\right)\\ X^{c+h}(t) & =X^{c+h}(0)+\sum_{j=1}^{M}\nu_{j}Y_{j,1}\left(\int_{0}^{t}m_{j,c,h}(s)\;ds\right)\\ & \;+\sum_{j=1}^{M}\nu_{k}Y_{j,3}\left(\int_{0}^{t}a_{j}^{c+h}(X^{c+h}(s))-m_{j,c,h}(s)\;ds\right) \end{array}$$

with $m_{j,c,h}(t)=\min\left\{a_{j}^{c}(X^{c}(t)),a_{j}^{c+h}(X^{c+h}(t))\right\}$. Here $Y_{j,1},\;Y_{j,2}$ and $Y_{j,3}$ are independent unit rate Poisson processes.

## 3 CIT method

In this section, we propose a new technique for approximating local sensitivities for stochastic discrete models of biochemical systems. This method is effective and accurate for stiff models (involving multiple scales in time). Stiff systems are often encountered in applications, as biochemical systems regularly involve both fast and slow reactions. In contrast with the CRN, CRP and CFD finite‐difference schemes, which use exact SSAs to generate the nominal and perturbed trajectories, our strategy computes coupled paths using the (approximate) implicit tau‐leaping strategy. The coupling we employ is related to [23], which is used in the CFD method [16]. This coupling shares similarities to the coupling in [24] and is applied in [20] for designing multi‐level Monte Carlo methods for well‐stirred stochastic biochemical systems. The CTL method for sensitivity [17] uses finite‐differences to estimate the sensitivities and the (approximate) explicit tau‐leaping strategy to generate the coupled trajectories. However, the CTL was designed for biochemical networks that are at most moderately stiff. As opposed to these approaches, the novel CIT technique involves solving implicit equations. For stiff to very stiff models, the proposed CIT strategy allows much larger time‐steps than the previous methods. Consequently, the CIT algorithm is expected to be significantly more effective that the existing finite‐difference estimators for such systems.

In the CIT algorithm, the coupled (i.e. nominal and perturbed) implicit tau‐leaping trajectories are generated as follows

(14) $$\begin{array}{rl} X^{c}(t+\tau ) & =x^{c}+\sum_{j=1}^{M}\nu_{j}[(a_{j}^{c}(X^{c}(t+\tau ))-a_{j}^{c}(x^{c}))\tau \\ & \;+P_{1,j}(m_{j,c,h}(x^{c},x^{c+h})\tau )\\ & \;+P_{2,j}((a_{j}^{c}(x^{c})-m_{j,c,h}(x^{c},x^{c+h}))\tau )] \end{array}$$

(15) $$\begin{array}{rl} X^{c+h}(t+\tau ) & =x^{c+h}+\sum_{j=1}^{M}\nu_{j}[(a_{j}^{c+h}(X^{c+h}(t+\tau ))\\ & \;-a_{j}^{c+h}(x^{c+h}))\tau +P_{1,j}(m_{j,c,h}(x^{c},x^{c+h})\tau )\\ & \;+P_{3,j}((a_{j}^{c+h}(x^{c+h})-m_{j,c,h}(x^{c},x^{c+h}))\tau )] \end{array}$$

with $X^{c+h}(t)=x^{c+h}$ and $X^{c}(t)=x^{c}$. The Poisson random variables $P_{1,j}$, $P_{2,j}$ and $P_{3,j}$ are independent. Here $m_{j,c,h}(x^{c},x^{c+h})=\min\left\{a_{j}^{c}(x^{c}),a_{j}^{c+h}(x^{c+h})\right\}$. The contribution of the shared term, $P_{1,j}(m_{j,c,h}(x^{c},x^{c+h})\tau )$, is expected to be significant, thus leading to a strong coupling. A consequence of this strong coupling is the tendency for reduced variance observed for this method (as illustrated in the next section). Once the Poisson terms are generated, Newton's method is applied to solve numerically both implicit equations: (14) for $X^{c}(t+\tau )$ and (15) for $X^{c+h}(t+\tau )$, respectively.

For advancing the numerical solution, the CIT utilises an extension of the adaptive time‐stepping strategy introduced by Cao *et al.* [19], for the implicit tau‐leaping method, as outlined in the previous section. A candidate leap is computed for the critical and non‐critical reactions, independently, on each of the nominal and perturbed trajectories, and then the smallest leap size is chosen as the next step.

*CIT algorithm*

1. *Set simulation parameters:* the tolerance for tau‐leaping ε, the tolerance for Newton's method, *TOL*, the critical threshold $n_{c}$, the final time *T* and the partial equilibrium parameter δ.
2. *Initialise sample paths:* the time t←0 and the states $X^{c+h}\leftarrow x_{0}$ and $X^{c}\leftarrow x_{0}$.
3. While *t* < *T*
  (a) *Compute propensity functions:* $a_{j}^{c+h}(X^{c+h})$ and $a_{j}^{c}(X^{c})$.
  (b) *Partial equilibrium condition:* for each set of reversible reactions in both systems, use (4) to check if they are in partial equilibrium.
  (c) *Find set of critical reactions for nominal and perturbed paths:* for each non‐partial equilibrium reaction $R_{j}$ in the two systems, with $a_{j}^{c}(X^{c})>0$ or $a_{j}^{c+h}(X^{c+h})>0$, compute
$$L_{j}=\underset{i\in [1,N];v_{ij}<0}{\min}\left\lfloor \frac{x_{i}}{\left|\nu_{ij}\right|}\right\rfloor ,$$
    where ⌊⋅⌋ is the ‘greatest integer in’ and set $J_{\text{ncr}}=\{j:L_{j}\geq n_{c}\}$.
  (d) *Compute candidate stepsizes,* $\tau_{1}^{\left(c\right)}$ *and* $\tau_{1}^{\left(c+h\right)}$ *, for non‐critical and not in partial equilibrium reactions:* If no non‐critical reactions occur, $\tau_{1}^{\left(c\right)}=\tau_{1}^{\left(c+h\right)}=\infty$. Else, determine $I_{\text{ncr}}$. For $i\in I_{\text{ncr}}$ on each of nominal and perturbed paths find:
    (I) $\psi_{i}$, the highest order at which $S_{i}$ appears in a non‐critical reaction.
    (II) $g_{i}$, the highest order at which species $S_{i}$ reacts.
    (III) ${\hat{\mu }}_{i}(x)$, ${\hat{\delta }}_{i}^{2}(x)$ using (7) and (8) and $\tau_{1}^{\left(c\right)}$, $\tau_{1}^{\left(c+h\right)}$ employing (6).
  (e) *Compute candidate stepsizes,* $\tau_{2}^{\left(c\right)}$ *and* $\tau_{2}^{\left(c+h\right)}$ *, for critical reactions:* calculate $a_{0}^{\text{cr},(c)}(X^{c})$, $a_{0}^{\text{cr},(c+h)}(X^{c+h})$, sample $\xi_{1}^{\left(c\right)}$, $\xi_{1}^{\left(c+h\right)}$ from U(0,1), and find $\tau_{2}^{\left(c\right)}$, $\tau_{2}^{\left(c+h\right)}$, using (10) and (12).
  (f) *Determine next stepsize and critical reaction index:* Let $\tau_{1}=\min\{\tau_{1}^{\left(c\right)},\tau_{1}^{\left(c+h\right)}\}$ and $\tau_{2}=\min\{\tau_{2}^{\left(c\right)},\tau_{2}^{\left(c+h\right)}\}$. Set $k_{j}^{c}=k_{j}^{c+h}=0$ for all critical reactions.
    (I) If $\tau_{1}^{\left(c\right)}<\tau_{2}^{\left(c\right)}$ and $\tau_{1}^{\left(c+h\right)}<\tau_{2}^{\left(c+h\right)}$, set $\tau =\tau_{1}$.
    (II) else if $\tau_{2}^{\left(c\right)}<\tau_{2}^{\left(c+h\right)}$, a sample $\xi_{2}$ from U(0,1). Choose $j_{cr}$ as smallest integer satisfying (9). Take $\tau =\tau_{2}$, $k_{j_{\text{cr}}}^{c}=1$.
    (III) else if $\tau_{2}^{\left(c+h\right)}<\tau_{2}^{\left(c\right)}$, a sample $\xi_{2}$ from U(0,1). Choose $j_{cr}$ as smallest integer satisfying (11). Take $\tau =\tau_{2}$, $k_{j_{\text{cr}}}^{c+h}=1$.
    (IV) else sample $\xi_{2}$ from U(0,1). Choose $j_{cr}$ as smallest integer satisfying (9). Take $\tau =\tau_{2}$, $k_{j_{\text{cr}}}^{c}=k_{j_{\text{cr}}}^{c+h}=1$.
  (g) *Step over non‐critical reactions:* For each $j\in J_{\text{ncr}}$,
$$m_{j}=\min(a_{j}^{c}(X^{c}),a_{j}^{c+h}(X^{c+h}))$$
    (I) Generate samples from Poisson distributions
$$\begin{array}{rl} P_{1,j} & =\text{Poisson}(m_{j}\tau ),\\ P_{2,j} & =\text{Poisson}((a_{j}^{c}(X^{c})-m_{j})\tau ),\\ P_{3,j} & =\text{Poisson}((a_{j}^{c+h}(X^{c+h})-m_{j})\tau ), \end{array}$$
    (II) Apply Newton's method to solve each of the systems
$$\begin{array}{c} U=X^{c}+\sum_{j\in J_{\text{ncr}}}\{[a_{j}^{c}(U)-a_{j}^{c}(X^{c})]\tau +P_{1,j}+P_{2,j}\}\nu_{j},\;\;\;\;\\ V=X^{c+h}+\sum_{j\in J_{\text{ncr}}}\{[(a_{j}^{c+h}(V)-a_{j}^{c+h}(X^{c+h})]\tau +P_{1,j}+P_{3,j}\}\nu_{j}. \end{array}$$
    (III) Update $X^{c}\leftarrow U$, $X^{c+h}\leftarrow V$.
  (g) *Implement the step:* update time t←t+τ and system states
$$\begin{array}{c} X^{c}\leftarrow X^{c}+\sum_{j\in J_{\text{cr}}}k_{j}^{c}\nu_{j},\;\;\\ X^{c+h}\leftarrow X^{c+h}+\sum_{j\in J_{\text{cr}}}k_{j}^{c+h}\nu_{j}. \end{array}$$
  (h) *Approximate sensitivity on the sample path:* $Z=(f(X^{c+h})-f(X^{c}))/h$ at the current time.

## 4 Numerical results

In this section, we compare the CIT method with the CFD strategy on some examples of stiff biochemical systems. Recall that, of the published finite‐difference techniques for estimating the sensitivities, the CFD technique provides estimates with the lowest variance [16].

In our comparisons, we use ensembles of 10,000 paths of the CFD and of the new CIT methods, respectively. We apply the CIT algorithm as described above with tolerance ε=0.05, TOL = 0.01, and δ=0.05. We show that the CIT method produces smaller variances than the CFD strategy for the first two models and similar variances for the third model. The CIT estimator is found to be significantly faster than the CFD. The efficiency is measured by

$$\text{Speed}‐\text{up}\;\text{over}\;\text{CFD}=\frac{\text{CPU}(\text{CFD})}{\text{CPU}(\text{CIT})},$$

where the CPU time to simulate 10,000 trajectories is considered in each case.

### 4.1 Decay‐dimerisation model

**Fig. 1:**
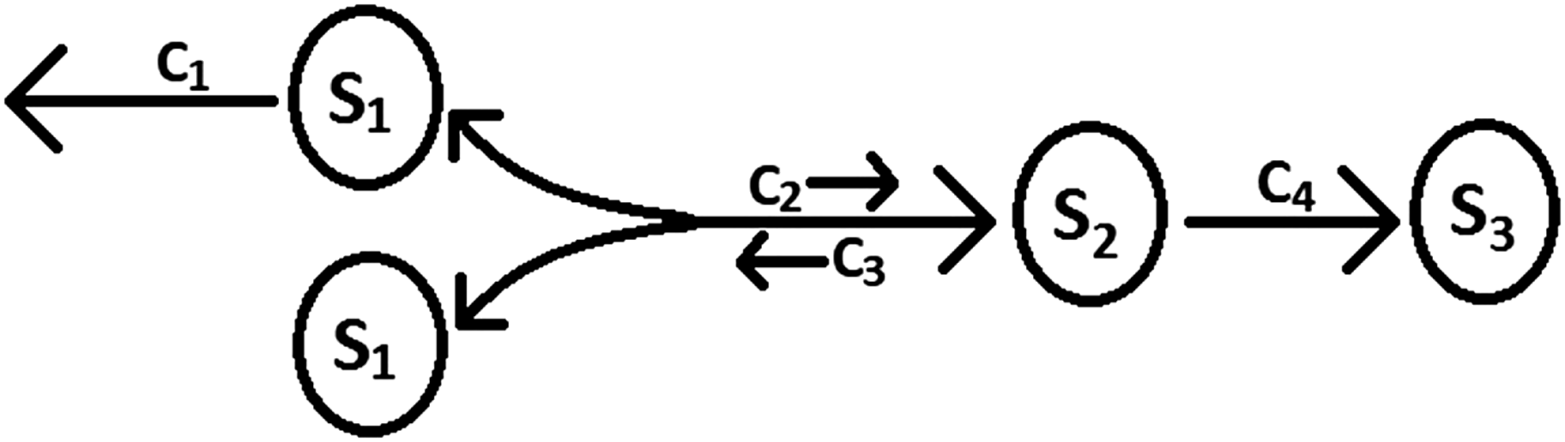
Decay‐dimerisation model reaction chain

The decay‐dimerisation model of [11] consists of three molecular species involved in four chemical reactions (Fig. 1). The reactions and propensities are given in Table 1, along with a set of nominal values for the rate constants.

**Table 1:** Decay‐dimerisation model

	Reaction	Propensity	Nominal rate constant
$R_{1}$	$S_{1}\overset{C_{1}}{⟶}⊘$	$a_{1}=C_{1}X_{1}$	$C_{1}=0.05$
$R_{2}$	$S_{1}+S_{1}\overset{C_{2}}{⟶}S_{2}$	$a_{2}=C_{2}X_{1}(X_{1}-1)/2$	$C_{2}=50$
$R_{3}$	$S_{2}\overset{C_{3}}{⟶}S_{1}+S_{1}$	$a_{3}=C_{3}X_{2}$	$C_{3}=1,000,000$
$R_{4}$	$S_{2}\overset{C_{4}}{⟶}S_{3}$	$a_{4}=C_{4}X_{2}$	$C_{4}=0.05$

**Fig. 2:**
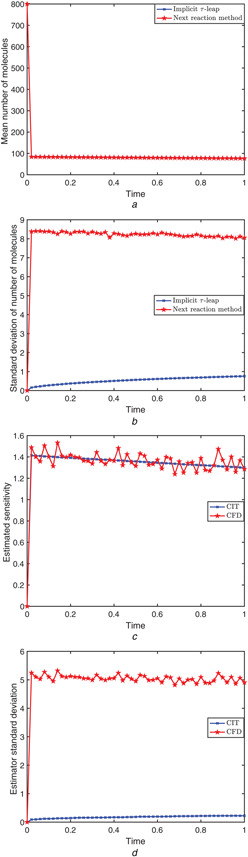
*Decay‐dimerisation model: 10,000 trajectories were generated on the time‐interval [0,1], with initial condition* $\left(X_{1}(0),X_{2}(0),X_{3}(0))=(400,800,0\right)$ *and parameters in Table* 1 ***(a)***, ***(b)*** Mean and standard deviation of the number of molecules for species $S_{2}$ were calculated by the next reaction method and the adaptive implicit tau‐leaping algorithm, ***(c)***, ***(d)*** Finite‐difference estimates of the sensitivity of the abundance of $S_{2}$ with respect to $C_{2}$, and the standard deviation of the estimators, for the CFD and CIT

The system was simulated on the time‐interval [0, 1], with initial conditions $\left(X_{1}(0),X_{2}(0),X_{3}(0))=(400,800,0\right)$ and the parameter $n_{c}=10$. The mean of the state variable $X_{2}$ (i.e. the number of $S_{2}$ molecules), for the adaptive implicit tau‐leaping algorithm and for the next reaction method (used for the CFD), are plotted in Fig. 2*a*. Fig. 2*b* shows the standard deviation of this state variable. The estimated sensitivity of $S_{2}$ with respect to the parameter $C_{2}$ and its standard deviation are shown in Figs. 2*c* and *d*. The perturbation parameter is *h* = 0.05 (i.e. 0.1% of the nominal parameter value). Fig. 2*d* demonstrates that the variance of the CIT estimator is small compared to that of the CFD, demonstrating accuracy. Moreover, the speed‐up of the CIT scheme over the CFD technique for estimating sensitivities for this particular simulation is

(16) Speed‐upoverCFD=9632.70.

### 4.2 Genetic positive feedback loop

**Fig. 3:**
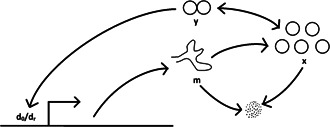
Schematic diagram of genetic positive feedback loop model

We next consider a simple model of positive feedback in gene expression (Fig. 3), as presented in [25]. Referring to Table 2, *x* represents a monomeric protein, *y* the protein dimer, $d_{0}$ the unoccupied regulatory site on the gene coding for *x*, $d_{r}$ the dimer‐occupied site, and *m*, the *mRNA* transcript. The reactions, propensities and a set of nominal parameter values are included in the table.

**Table 2:** Genetic positive feedback loop model

	Reaction	Propensity	Nominal rate constant
$R_{1}$	$x+x\overset{C_{1}}{⟶}y$	$a_{1}=C_{1}X(X-1)/2$	$C_{1}=5000$
$R_{2}$	$y\overset{C_{2}}{⟶}x+x$	$a_{2}=C_{2}Y$	$C_{2}=10^{6}$
$R_{3}$	$y+d_{0}\overset{C_{3}}{⟶}d_{r}$	$a_{3}=C_{3}YD_{0}$	$C_{3}=5000$
$R_{4}$	$d_{r}\overset{C_{4}}{⟶}y+d_{0}$	$a_{4}=C_{4}D_{r}$	$C_{4}=10^{6}$
$R_{5}$	$d_{0}\overset{C_{5}}{⟶}d_{0}+m$	$a_{5}=C_{5}d_{0}$	$C_{5}=10$
$R_{6}$	$d_{r}\overset{C_{6}}{⟶}d_{r}+m$	$a_{6}=C_{6}D_{r}$	$C_{6}=20$
$R_{7}$	$m\overset{C_{7}}{⟶}m+x$	$a_{7}=C_{7}M$	$C_{7}=1$
$R_{8}$	$x\overset{C_{8}}{⟶}⊘$	$a_{8}=C_{8}X$	$C_{8}=0.8$
$R_{9}$	$m\overset{C_{9}}{⟶}⊘$	$a_{9}=C_{9}M$	$C_{9}=7$

We ran simulations from initial molecular amounts of $\left(X_{1}(0),X_{2}(0),X_{3}(0),X_{4}(0),X_{5}(0))=(10,20,10,40,0\right)$ over the time‐interval [0,2], with $n_{c}=10$.

**Fig. 4:**
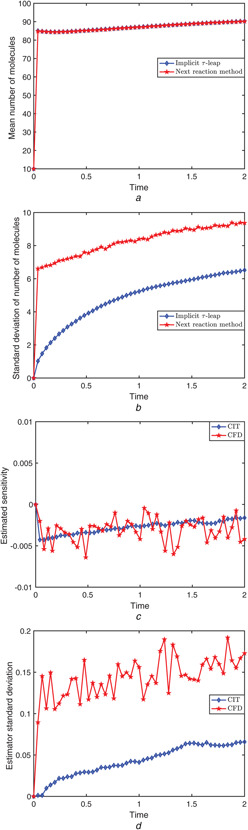
*Genetic positive feedback loop model. 10,000 sample paths with initial condition* $\left(X_{1}(0),X_{2}(0),X_{3}(0),X_{4}(0),X_{5}(0))=(10,20,10,40,0\right)$ *and parameters as given in Table* 2 *were generated on the time‐interval [0,2]* ***(a)***, ***(b)*** Mean and standard deviation of the number of molecules for species *x* were calculated by the next reaction method and the adaptive Implicit tau‐leaping algorithm, ***(c)***, ***(d)*** Mean and standard deviation of the finite‐difference estimators determined via the CFD and implicit tau‐leaping methods, of the sensitivity of the abundance of *x* to the parameter $C_{1}$

Fig. 4*a* presents the evolution of the mean amount of the *x* molecules over 10,000 paths, generated with the CIT algorithm and the next reaction method, respectively. The standard deviation of the molecular count of *x* as a function of time, for each of the two algorithms, is shown in Fig. 4*b*. The behaviours of the estimated sensitivity of the *x* molecular numbers with respect to the parameter $C_{1}$, using the CIT and the CFD methods are presented in Fig. 4*c*, whereas the corresponding standard deviations of the CIT and CFD estimators are given in Fig. 4*d*. The simulations are performed with a perturbation *h* = 0.5 (i.e. 0.01% of the nominal parameter value). From Fig. 4*d*, we observe that the CIT estimator variance is low compared to the variance of the CFD estimator, therefore the sensitivity estimation of the new CIT method is more accurate. This low variance is confirmed as shown in Fig. 4*c*. For the set of parameters used, the speed‐up, on time interval [0, 2], of the CIT over the CFD is significant

(17) Speed‐upoverCFD=2656.43.

### 4.3 Collins toggle switch model

**Fig. 5:**
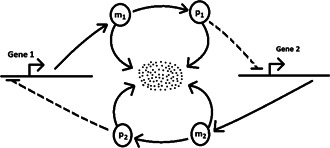
Collins toggle switch model reaction scheme diagram

**Fig. 6:**
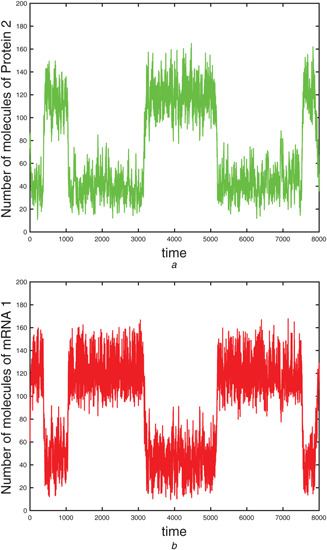
*Collin's toggle switch model: a sample path of species* $p_{2}$ *and* $m_{1}$, *with initial condition* $\left(X_{1}(0),X_{2}(0),X_{3}(0),X_{4}(0))=(76,75,60,60\right)$ *and the parameters in Table* 3 *generated with the implicit tau‐leaping method on the time‐interval [0, 8000]*

**Fig. 7:**
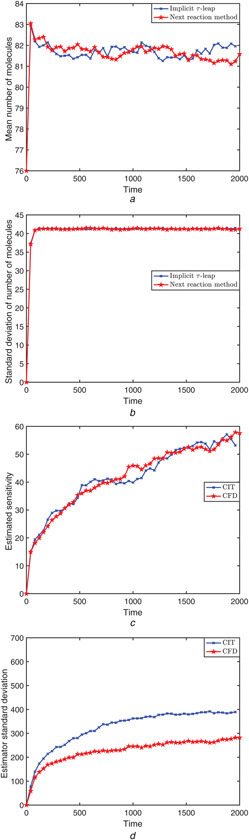
*Collins toggle switch model. 10,000 sample paths with initial condition* $\left(X_{1}(0),X_{2}(0),X_{3}(0),X_{4}(0))=(76,75,60,60\right)$ *and parameters as given in Table* 3 *were generated on the time‐interval [0, 2000]* ***(a)***, ***(b)*** Mean and standard deviation of the number of molecules for species $p_{1}$ were calculated by the next reaction method and the adaptive Implicit tau‐leaping algorithm, ***(c)***, ***(d)*** Mean and standard deviation of the finite‐difference estimators determined via the CFD and implicit tau‐leaping methods, of the sensitivity of the abundance of $p_{1}$ to the parameter $C_{1}$

The Collins toggle switch [26] is a gene regulatory network that exhibits bistability: it consists of two genes, each encoding a repressor of the other. Referring to Fig. 5, the species $p_{1}$ and $p_{2}$ are the gene's protein products, while $m_{1}$ and $m_{2}$ denote the corresponding *mRNA* transcripts. The parameters $\alpha_{1}$ and $\alpha_{2}$ denote the maximal transcription rates. Furthermore, β and γ are the degrees of non‐linearity in the repression mechanisms. The system exhibits bistable‐like behaviour when $\alpha_{1}≃\alpha_{2}$ and the maximal expression rates are adequately large. We introduce a scaling factor *k* on the propensity of mRNA transcription and degradation to allow tuning of the model stiffness. For Figs. 6 and 7, we used *k* = 1000. Table 3 lists the reactions, their propensities and nominal rate constants. This illustrative parameterisation was chosen to achieve regular transitions between well‐separated quasi‐steady states and significant stiffness.

**Table 3:** Collins toggle switch model

	Reaction	Propensity	Nominal rate constant
$R_{1}$	$⊘\overset{C_{1}}{⟶}m_{1}$	$a_{1}=k\frac{\alpha_{1}}{1+{\left(X_{2}\right)}^{\beta }}$	$\begin{array}{rl} \alpha_{1} & =C_{1}=28.98\\ \beta & =4 \end{array}$
$R_{2}$	$m_{1}\overset{C_{2}}{⟶}⊘$	$a_{2}=kC_{2}X_{3}$	$C_{2}=0.23$
$R_{3}$	$m_{1}\overset{C_{3}}{⟶}p_{1}+m_{1}$	$a_{3}=C_{3}X_{3}$	$C_{3}=0.23$
$R_{4}$	$p_{1}\overset{C_{4}}{⟶}⊘$	$a_{4}=C_{4}X_{1}$	$C_{4}=0.23$
$R_{5}$	$⊘\overset{C_{5}}{⟶}m_{2}$	$a_{5}=k\frac{\alpha_{2}}{1+{\left(X_{1}\right)}^{\gamma }}$	$\begin{array}{rl} \alpha_{2} & =C_{5}=28.98\\ \gamma & =4 \end{array}$
$R_{6}$	$m_{2}\overset{C_{6}}{⟶}⊘$	$a_{6}=kC_{6}X_{4}$	$C_{6}=0.23$
$R_{7}$	$m_{2}\overset{C_{7}}{⟶}p_{2}+m_{2}$	$a_{7}=C_{7}X_{4}$	$C_{7}=0.23$
$R_{8}$	$p_{2}\overset{C_{8}}{⟶}⊘$	$a_{8}=C_{8}X_{2}$	$C_{8}=0.23$

This system was integrated on the time‐interval [0, 2000], with initial conditions X(0)=(76,75,60,60) and $n_{c}=5$. Sample trajectories for the species $p_{2}$ and $m_{1}$, simulated with the underlying implicit tau‐leaping method are shown in Figs. 6*a* and *b*. Bistable behaviour is also observed for the species $m_{2}$ and $p_{1}$ (not shown). The mean and standard deviation number of the number of $p_{1}$ molecules for the implicit tau‐leaping algorithm and for the next reaction method are plotted in Figs. 7*a* and *b*, respectively. We remark that, for this bistable model, the variance of the implicit tau‐leaping technique matches well the variance of the (exact) next reaction method, in contrast with the behaviour of the implicit tau‐leaping scheme observed by Rathinam *et al.* [11] for systems reaching a steady state, where the implicit scheme reduced the variance of the numerical solution. Figs. 7*c* and *d* present the finite‐difference estimation of the sensitivity of the $p_{1}$ molecule count with respect to the parameter $C_{1}$ and the estimator's standard deviation for each of the CIT and CFD algorithms. In these simulations, the perturbation parameter is *h* = 0.05 (i.e. 0.2% of the nominal parameter value). The estimation of the sensitivity is similar for the CIT and the CFD methods, while the standard deviation of the CIT estimator is slightly larger than that of the CFD estimator. However, for the set of parameters in Table 3, the speed‐up of the CIT over the CFD is 74‐fold.

To gauge the performance of the method as stiffness increases, the performance of the CIT and CFD methods was studied for various values of scaling parameter *k*. The chosen range produced stiff to very stiff model formulations, resulting in speed‐up of the new CIT strategy compared to the existing CFD method of up to 468 times, as reported in Table 4. For non‐stiff models, the CIT algorithm will perform no better than the CFD method. For this model, a similar computational time for the two algorithms is obtained when the propensities of the fastest and slowest reactions are separated by about two orders of magnitude.

**Table 4:** Collins toggle switch model: the speed‐up of the CIT compared to the CFD for estimating the sensitivity of $p_{1}$ with respect to $C_{1}$ for *h* = 0.05 on time interval [0, 2000]

Method	Stiffness parameter *k*	Speed‐up
CIT	300	10.16
CIT	1000	74.71
CIT	3000	468.43
CFD	—	1

As shown in panel (d) of Figs. 1, 4 and 7, the variance of the CIT estimator is not always comparable to that of the CFD estimator (smaller in the first two examples, larger in the third). For first two models, we observe that our CIT method is more accurate and far more efficient than the existing CFD strategy.

For the third model, when the value of stiffness parameter *k* grows, our CIT method becomes increasingly more efficient than the CFD scheme. On the other hand, for the Collins toggle switch model, the variance of the CIT estimator is slightly larger than that of the CFD. The implicit tau‐leaping scheme damps the noise for systems reaching a steady state [11]. However, for the toggle switch model, the implicit tau‐leaping scheme does not cause noise reduction. Trajectories frequently switch between two states, the model exhibiting bistable behaviour. This behaviour restricts the noise damping property of the implicit tau‐leaping scheme and leads to a slightly larger variance of the CIT algorithm than that of the CFD, unlike for the previous two models. According to our numerical experiments, we conclude that our CIT method is expected to be more accurate and significantly more efficient than the CFD technique when the stiff system reaches a steady state. A theoretical study of the properties of the finite‐difference CIT sensitivity estimator and appropriate values of the perturbation parameter *h* leading to optimal convergence rates will be considered in our future work.

## 5 Conclusion

Noise plays an important role in the behaviour of well‐stirred biochemical systems when some species have low molecular counts. Usually, such systems are represented using the discrete stochastic CME model. For the class of biochemical systems that are mathematically stiff, implicit τ‐leaping schemes are preferred over exact Monte Carlo SSAs; implicit methods are considerably more efficient for accurately determining the slow stochastic variables of the system, and for determining the mean behaviour of the fast variables. Indeed, the implicit tau‐leaping strategy allows large stepsizes while maintaining the solution close to the slow manifold. By contrast, exact stochastic simulation techniques are forced to take very small time‐steps when stiffness is encountered.

This work developed a new finite‐difference estimator of sensitivities for stochastic discrete models of biochemical kinetics. The new sensitivity estimator, the CIT or CIT, employs an adaptive implicit τ‐leaping method to simulate the nominal and perturbed paths. To enhance the accuracy of the estimation, the CIT applies a strong coupling between the nominal and perturbed trajectories. The new sensitivity estimator is superior to Anderson's CFD estimator [16] for models of biochemical systems that are stiff to very stiff. We demonstrated that the CIT has a considerably reduced computational cost compared to the CFD while retaining an equivalent accuracy. Thus, for the class of stiff to very stiff biochemical reaction networks, the coupled implicit τ‐leaping method is a preferred choice for sensitivity analysis.
